# Rapid migration of CO_2_-rich micro-fluids in calcite matrices

**DOI:** 10.1038/s41598-018-32461-8

**Published:** 2018-09-20

**Authors:** Pierpaolo Zuddas, Stefano Salvi, Olivier Lopez, Giovanni DeGiudici, Paolo Censi

**Affiliations:** 1 Sorbonne Université, CNRS, ISTeP, 4 place Jussieu, 75005 Paris, France; 2 GET, Université de Toulouse, CNRS, IRD, CNES, Toulouse, France; 3 Statoil, Trondheim, Norway; 4 Department of Chemistry and Earth Sciences, University of Cagliari, Cagliari, Italy; 5 DISTEM, University of Palermo, Palermo, Italy

## Abstract

The transport of supercritical fluids is a determining factor for several geological processes and fundamental in predicting natural resource accumulation and distribution. Calcite, ubiquitous in most geological environments, may contain supercritical CO_2_ trapped under the form of fluid inclusions that may move through grain boundaries affecting the rock physical properties. However, despite macroscopic evidence for this process, until recent it was not possible to characterize this process at the nano-scale due to the difficulty of such observations. In this study, we report nanometer-scale observations on calcite crystal surfaces and demonstrate that stress with absence of visible deformation produces fluid leakage from fluid inclusions. Atomic Force Microscopy scanning experiments on freshly cleaved calcite crystals containing visible fluid inclusions revealed the spontaneous formation of nanometer-scale hillocks on flat crystal terraces in only a few minutes, without evidence of surface dissolution. The fact the hillocks formed on flat surface in a short time was unexpected and suggests deposition of material from the inner crystal to the surface through small-scale fluid migration. We estimated the rate of this fluid mobility is by several orders of magnitude higher than the diffusion rate through vacancies estimated in calcite crystals showing that CO_2_–rich fluids through micro-pore and nano-pore spaces is in reality much higher than previously assumed using current predictive models.

## Introduction

Fluids play a key role in enhancing mineral reactions and rock deformation, influencing a wide range of geological processes such as fluid-rock interaction, solution transfer, electrical conductivity, seismic wave velocity and attenuation^1^. Observations of minerals in a rock at high magnification has revealed that tiny aliquots of these paleofluids can be retained in microscopic cavities that form fluids inclusions wetting grain surfaces and forming a thin, continuous film that may short-circuit transport. In non-fractured rocks, however, fluids can easily move as they are located at the grain boundary spaces^2^. One significant weakness in our fluid transport knowledge concerns the mechanisms controlling the fluid distribution among grains of solid rock with which they are in chemical and mechanical equilibrium. The fluid flow between grains and mineral pores has been documented at elevated temperatures and pressures^3^ while it is assumed to be negligible at 25 °C and 1 atmosphere^4^. Some earlier evidence to the contrary is, however, constituted by the self-diffusion of calcium isotopes documented in some soil carbonates^5^. In the case of CO_2_-bearing aqueous fluids, the extreme conditions required for studying displacement processes at the micrometric and nanometric scale (i.e. the scale of pores and grain boundaries), are such that only a limited number of studies have been published on the subject^6,7^. These small-scale phenomena, interpreted as interfacial processes, have been often neglected in continuum-scale modelling such a viscous fingering^6^ and wetting alteration by CO_2_-rich fluids^7,8^. The rheological properties of thin complex fluids is of paramount importance for gas flow in rock matrices^9^ and geophysical models of non active tectonic areas^10,11^. Earlier work^12–14^ suggests that fluids confined at the micrometric (or nanometric) scale may move fast and have a key role in fluid-solid reactivity. Fluid movement and solid permeability at grain boundaries have often been explained in terms of interfacial energy^7^, while when a small amount of fluid is trapped by surface tension, the capillarity pressure is sheared. At this scale, fluids must be present in the form of absorbed molecular films, non-equilibrium or wetting film, as well as isolated fluid inclusions maintained by steric force originated from hydrated layer at the mineral surface^13,15^.

In this contribution, we report nanometer-scale observations made by Atomic Force microscopy (AFM) on natural calcite crystals containing micrometric fluid inclusions and demonstrate stress-induced production of fluid leakage from what most likely are nanometric fluid inclusions, in a very short time under standard temperature and pressure conditions. Our results yield the first independent determination of nano-scale fluid movement inside a calcite matrix. The potential significance of the results to fluid transport in rock is also briefly discussed. This study points the way for future similar research on other minerals, helpful for modelling fluid transport of matter, fault lubrication during earthquakes^16^ and geological sequestration of CO_2_^17^.

## Results

### Heterogeneity of natural calcite

**Figure 1:**
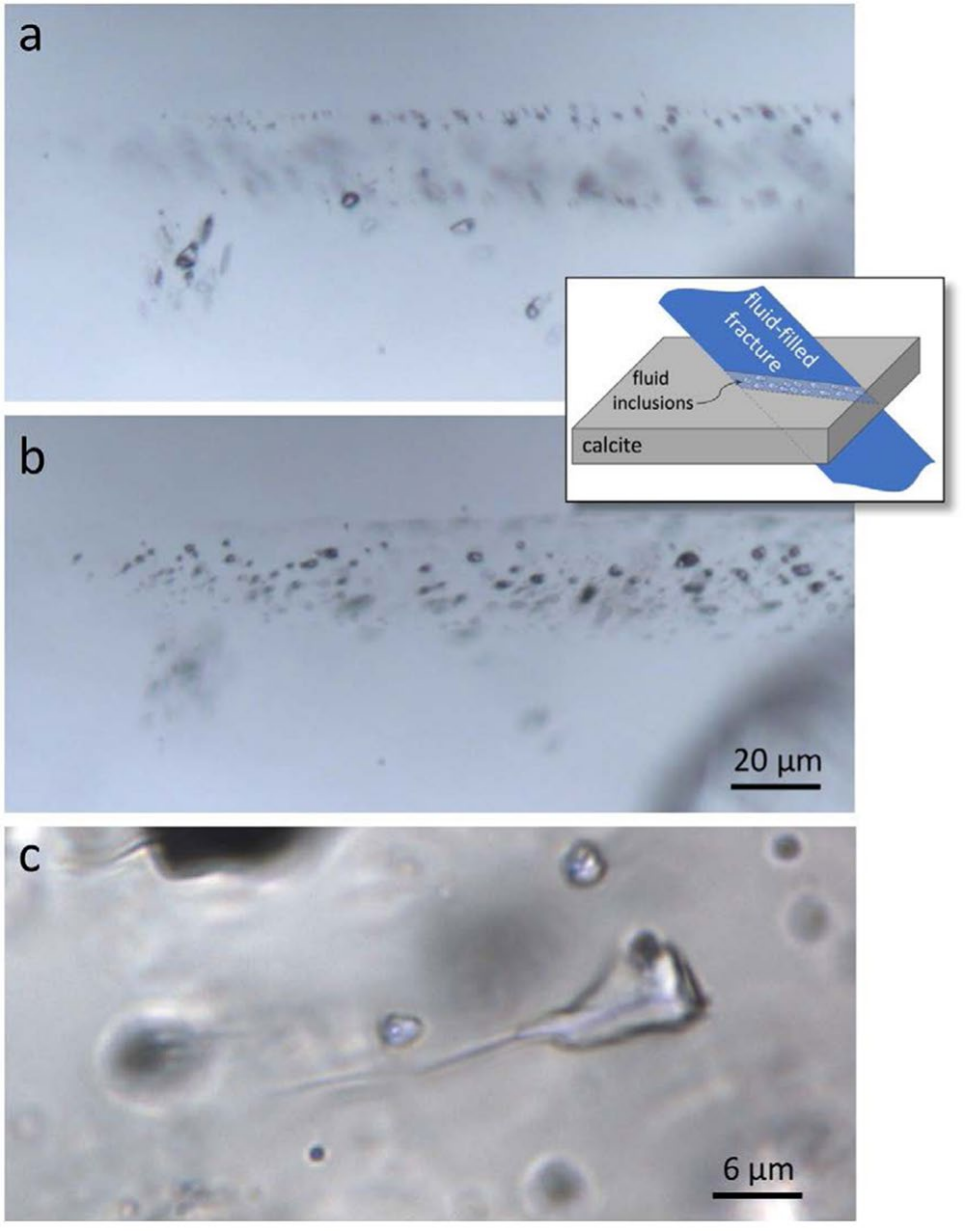
Optical microscope photomicrographs (plane polarized transmitted light) of a 120-µm-thick doubly-polished wafer (120-µm in thickness) prepared from a calcite Iceland spar. Images (**a**,**b**) show the same area at different focal depths (**a** nearest the surface and **b**: a few microns below) to illustrate a healed fracture defined by a plane of fluid inclusions (FI). The sketch in the inset illustrates the FI trapping mechanism. Note somewhat larger fluid inclusions off the plane. Reflection of internal features is due to the double refraction of calcite. Image (**c**) depicts a micrometric fluid inclusion in a crystal, showing a hairline fracture departing from it due to (partial) thermal decrepitation of the inclusion.

We monitored the state of a freshly cleaved calcite surface using optical-quality Iceland spar from Chihuahua, Mexico (99.5% CaCO_3_). Optical microscope observations of the calcite crystal denoted the presence of several fracture planes of few millimeters in length, as well as fluid inclusions organized along similar planes or occurring isolated or in small groups (Fig. 1a,b). The size of visible fluid inclusions ranges from <1 micron to relatively large (~20 micron), with shapes varying from a rhombohedrum to irregular morphology. Based on the number of phases present at room temperature and on microthermometric data, we identified two types of fluid inclusion: Type-1 fluid inclusions contain liquid H_2_O and a relatively high-density homogeneous CO_2_ phase (in a few fluid inclusions, CO_2_ consisted of distinct liquid and vapor phases); Type-2 fluid inclusions, in addition to aqueous and carbonic phases, contain halite and, in a few cases, other unidentified solids. Both fluid inclusion types occur together in planes and groups. On cooling, the homogeneous CO_2_ separated into liquid and vapor phases that, upon heating, homogenized to the liquid phases at temperatures ranging from −5° to 27 °C. The corresponding bulk density ranging from 0.85 to 1.15 g/cm^3^, which would produce internal pressures as high as 50 MPa at room temperature. The nature of dissolved salts in the aqueous solutions of both inclusion types, estimated by microthermometry (see method section), consists mostly of Na^+^ with salinity in the range of 5–26 wt. % NaCl equivalent. Some halite-bearing inclusions have higher salinity, the exact degree of which could not be determined because most inclusions decrepitated before total homogenization upon heating, likely due to high CO_2_ pressure. High Resolution Transmission Electron Microscopy observations reveal the presence of rupture of the periodicity of calcite producing several grain junctions delimiting variable size of mono-crystal domains ranging from 10 to 500 nm in diameter.

### AFM observation and evidences for fast fluid movement at standard conditions

**Figure 2:**
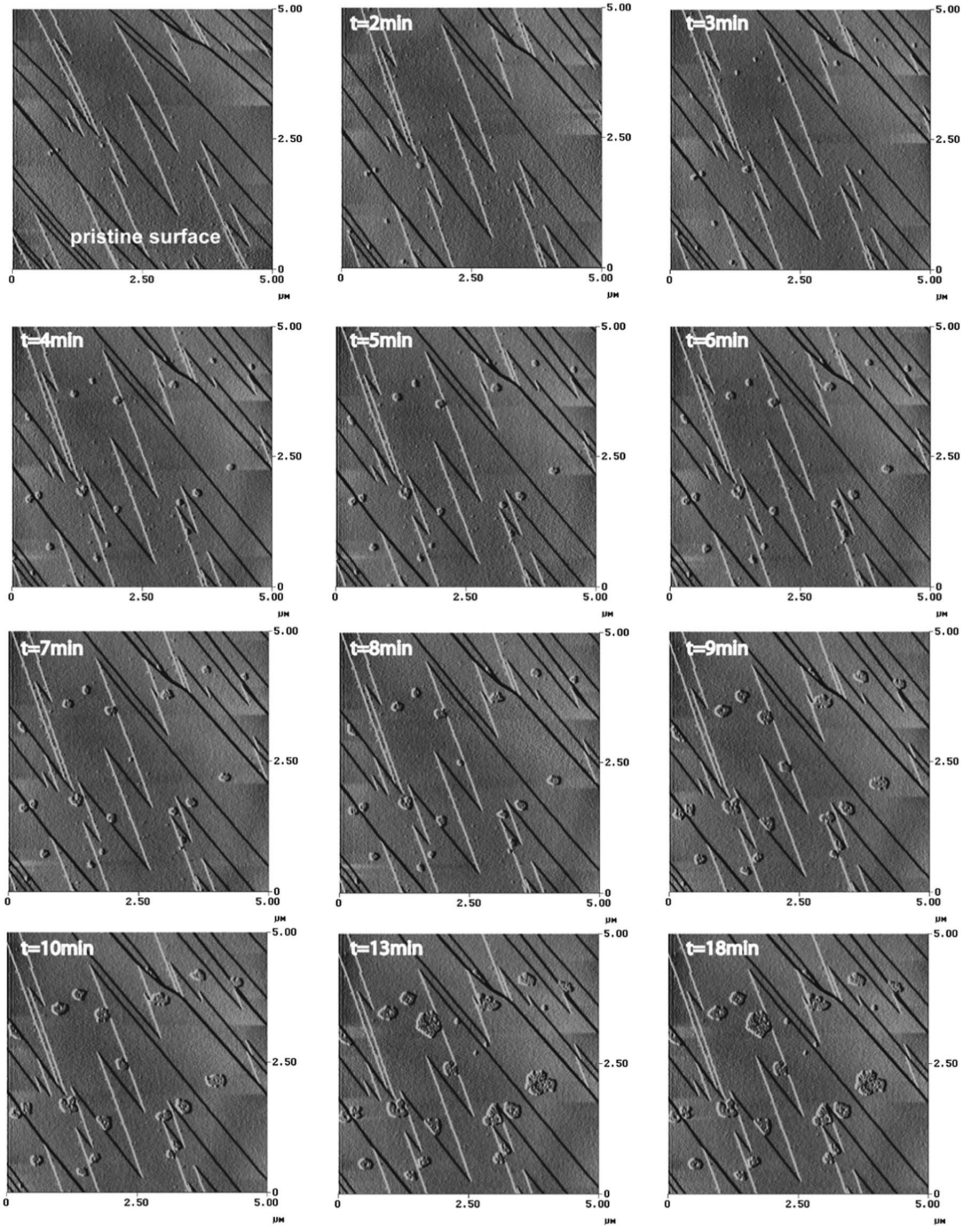
Evolution of the state of the $(10\bar{1}4)$ calcite surface during 18 min of AFM scanning at a scan frequency of 6 Hz and an applied force of 7 nN. The scan surface area is 5 *5 µm. After 2 min of scanning, randomly distributed hillocks appear over the investigated surface area. The average height is 9 ± 2 nm while size and geometry change. After 13 minutes of scanning, the growth stops.

Examination of over 100 freshly cleaved calcite surfaces by AFM showed the presence of atomically flat terraces separated by cleavage steps of various heights. During real-time monitoring of samples, after only two minutes of scanning, we observed nanometer-scale hillocks forming spontaneously on the calcite surface. These mounds did not concentrate on surface imperfection sites such as steps or kinks, which are recognized sites of excess energy, but appeared to be randomly distributed. Also, although hillocks grew with a preferential horizontal direction, they did not present any particular morphology or crystallographic pattern (Fig. 2). Their height averaged around 0.9 ± 0.2 nm, whereas their horizontal size and geometry varied slightly (0.01 to 0.5 µm). After 13 minutes of scanning, growth of the earliest-formed hillocks stopped, however, within this time range new hillock nuclei had appeared at other sites on the observed mineral surface. In this study 80% of the observation reflect the feature reported in Fig. 2. The fact that hillocks formed spontaneously on flat terraces in only few minutes, without evidence of surface dissolution, and under standard atmospheric conditions, was unexpected.

In an earlier study^18^ hillocks appeared only after 2–5 hours of AFM scanning on a mineral surface that presents evidence of dissolution pits. Changes of the cleaved calcite surface have also been observed in several X-ray reflectivity studies of air-calcite interaction^19,20^ and were explained by the absorption of water molecules coming from the surrounding air moisture^21^. However, our AFM experiments put into question the fact that the origin of the water molecules is exclusively due to the dampness in the air. The extreme rapidity of the phenomenon that we observed, and the absence of dissolution pits, eliminates the possibility that hillocks formed by dissolution/re-precipitation of small parts of the calcite surface. In addition, hillock growth was observed exclusively in the surface areas that were investigated, suggesting a possible role of internal characteristics of our calcite sample. We therefore set out to test the hypothesis that fluid inclusions of sub-micron size and located a few microns below the calcite surface are the source of at least some of this matter. In our samples, fluid inclusions consist of an aqueous solution enriched in Cl^−^ and Na^+^, coexisting with liquid CO_2_, confined at an internal pressure estimated at around 50 MPa. The strength of engagement of the AFM tip under our experimental conditions is by several orders of magnitude lower than the calcite Shear Modulus (modulus of rigidity measuring the stiffness of calcite macroscopic crystals, which is about 10^10^ Pa)^22^. The surface free energy of ‘dried’ large calcite crystals is equal to 0.59 J/mol, whereas that of ‘hydrated’ crystals is 0.33 J/mol^23^ and may decrease by one order of magnitude for smaller nanometric sized particules^24^. Assuming that the fluid inclusions are in equilibrium with the mineral walls, the stress produced by the tip could be responsible for the disruption of such conditions. Thus, sub-micron fluid inclusions can move through the calcite crystal (via dislocations) to the mineral surface and cause the formation of the observed hillocks. Migration of several-micron-sized fluid inclusion has been postulated by several authors in minerals such as quartz, via dislocation or by dissolution-precipitation of the crystal walls^25,26^.

### Estimating the fast micro-fluid movement

**Figure 3:**
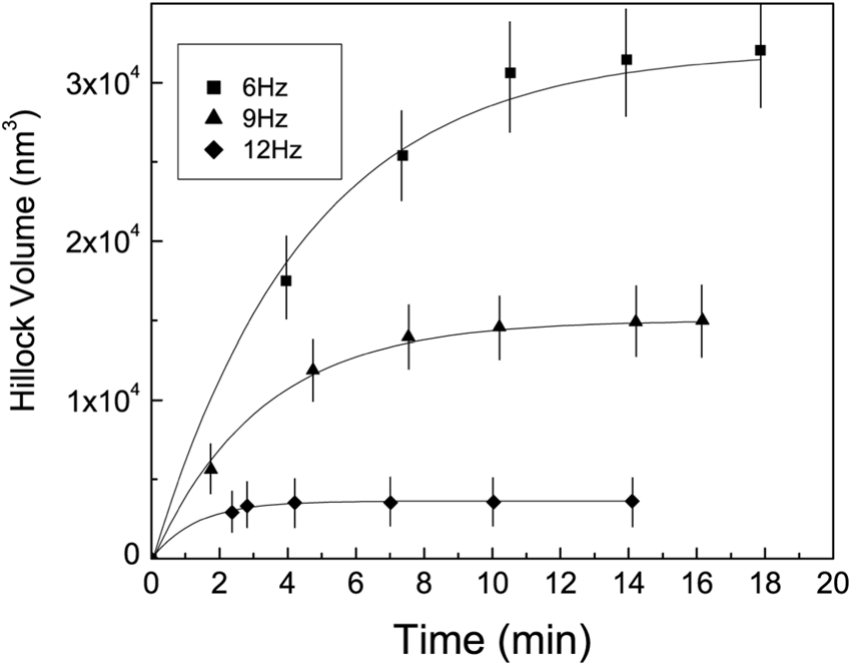
Evolution of the hillock volume as a function of time at different scan frequencies (in Hz). At lower frequencies, hillocks grow rapidly during the first 10 min. This fast growth rate decreases at higher scan frequencies. Lower AFM scan frequencies correspond to a higher time of contact between tip and surface.

**Figure 4:**
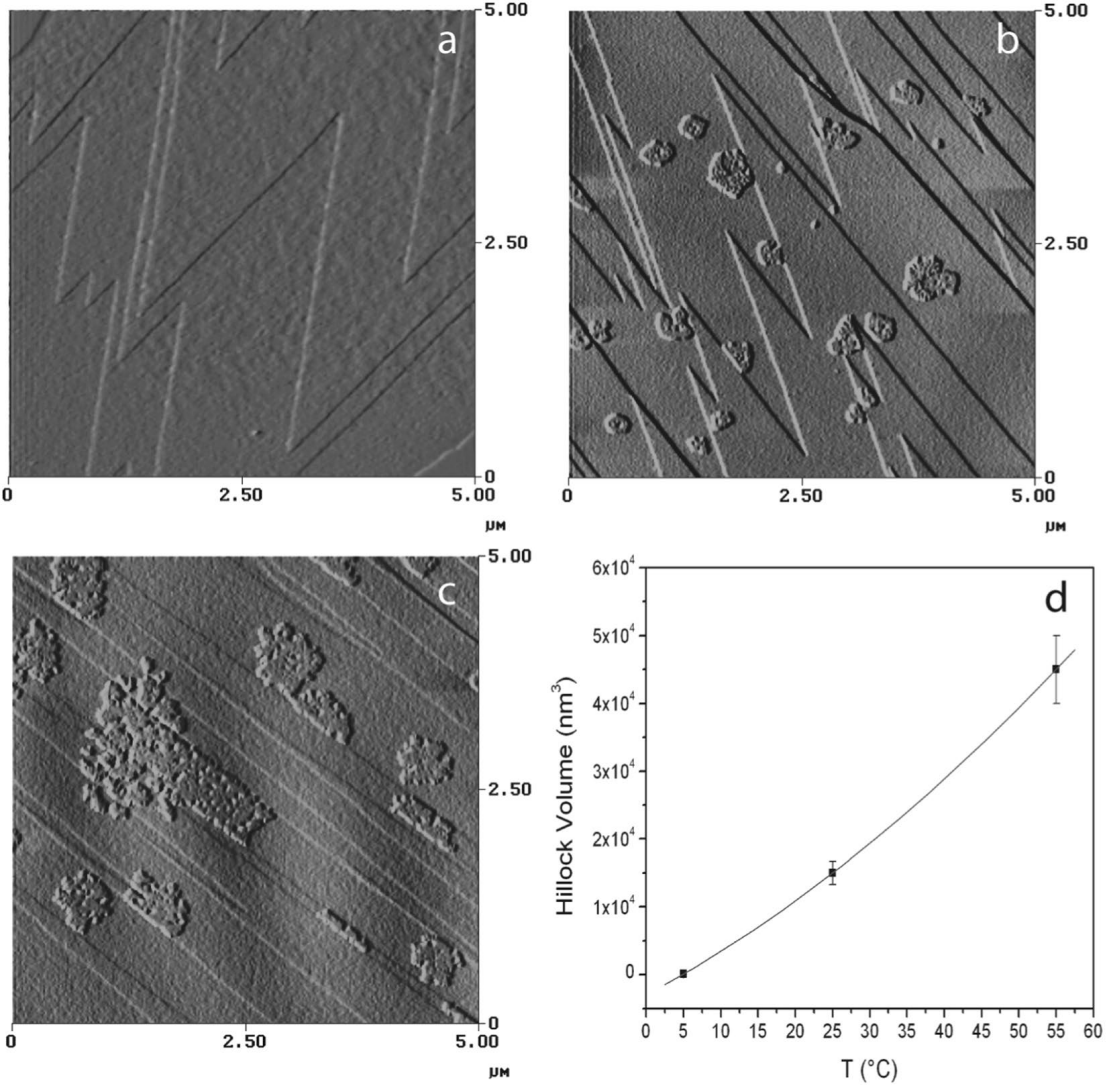
AFM images of a freshly cleaved calcite surface at different temperatures (**a**, 5 °C, **b**, 25 °C and **c**, 55 °C) obtained with a scan frequency of 9 Hz. (**d**) Represents the hillock volume formed after one hour of scanning.

We carried out a number of additional experiments to investigate the interaction between the AFM tip and the mineral surface. Since scan frequency of an AFM tip over a surface is inversely proportional to the tip-surface residence time, by decreasing the scan frequency we could increase the contact time (for a same scanned surface), thus enhancing the mechanical strain undergone by the mineral. Resulting data shows that a 50% decrease in scan frequency produced a 600% increase in hillock volume (Fig. 3). Moreover, hillock growth stopped more rapidly with higher scan frequencies. We also tested the influence of temperature by varying this parameter in the AFM cell from 5 to 50 ± 4 °C, and found that hillock volumes increased in high temperature runs, within similar observation time spans. We evaluated the volume variation, by estimating the dimensions of discrete feature in the AFM images^27^ and found that a 30 °C temperature increase produced a 4-fold increase in hillock volume (Fig. 4). At a temperature of 5 °C, however, hillocks did not form despite several hours of scanning. These results indicate that mechanical strain controls hillock formation and that this process is strongly enhanced at higher temperatures.

The observed hillock formation can be explained by the fact that CO_2_-rich fluid inclusions located just under the surface are near their rupture limit because of the elevated internal pressure, thus even a very small mechanical strain will cause them to decrepitate and lose their fluid. This phenomenon is potentially enhanced by heat, as any temperature increase would cause the internal pressure to rise dramatically due to conservation of internal density^28^. Fluid inclusion decrepitation in response to heat-induced overpressure is a well-known phenomenon in fluid-inclusion studies; hairline fracture propagating from the decrepitated micrometric fluid inclusions can sometimes be observed (cf. Fig. 1c). Alternatively, it is possible that fluid movement to the surface took place along preferential pathways such as micro or nano-fractures already present^29,30^. However, this would not be consistent with the fact that hillocks were randomly distributed over the calcite surface. Assuming that (i) the fluid inclusions involved in the fluid movement are located at least 0.01 to 0.1 µm below the calcite surface, (ii) the pathway is vertical, and (iii) precipitation at the surface is instantaneous, our real-time measurements allow us to estimate a rate of fluid transport at ~5 × 10^−10±0.5^ m·s^−1^. This value does not represent a diffusion coefficient in the sense of the Ficks Law but is simply an estimation of the rate of inner fluid-mobility in our calcite matrix^30^. The transport rate that we calculated is about 4–5 orders of magnitude higher than that estimated by low-temperature extrapolation of solid-state diffusion vacancy experiments^31^. These results highlight the extreme mobility of fluids at the micro-nano fluidic scale in calcite crystals at standard pressure and temperature conditions.

AFM observations alone cannot provide information on the composition of these surface features. However, the fact that the hillocks grow on the surface may correspond to an eventual accumulation by epitaxial growth^32^. The CO_2_ coming from the fluid inclusions could mix with the thin water layer in equilibrium with ambient air at the surface^33^, inducing precipitation of calcium carbonate hillocks. Alternatively, these hillocks could consist of halite nanocrystals resulting from evaporation of the NaCl-rich aqueous fluid coming from fluid inclusions. However, halite precipitates formed from heat-induced decrepitation of fluid inclusions generally display varied morphologies, ranging from cubic crystals to toothpaste-like tubes^28^, features that were not recognized in this study. We also carried out additional experiments under dry atmosphere conditions (using toluene) and found much smaller hillocks, suggesting the importance of ambient water in the formation of the hillocks and thus validating the former hypothesis.

### Interfacial transport at the micro-fluidic scale

Our experimental observations show that stress applied to a calcite crystal can produce fluid migration, at least within the more external parts of the crystal. The question that arises, then, is the limiting conditions for fluid mobility and when it could be a dominant transport mechanism. In a stable fluid inclusion, the liquid resistance to driving forces increases when the channel size decreases^13^. However, in case of micrometric or nanometric channel sizes, transport coefficients should take interfacial properties into account^34,35^. For simple fluid/solid interface with pressure flow represented by a slit of thickness h and slip length b, the mean velocity increases by a factor 1 + 6b/h as compared to the no-slip surfaces, suggesting that the size of the channel h drives the flow. However, our observations suggest that the interfacial structure at the solid-liquid interface could be an alternative way to generate flow. The electrical interaction between tip and surface atoms generates a stress over the mineral surface. Applying a stress E, the fluid acquires a plug flow comparable to a velocity profile, with a velocity ν_EO_ proportional to E in agreement with the Smoluchowski equation^34^:

1 $${\rm{\nu }}{{\rm{E}}}_{o}=-\,{\rm{\varepsilon }}{\rm{\zeta }}{\rm{E}}/{\rm{\eta }}$$

where ε is the dielectric permittivity of the solvent; η its viscosity; ζ is the Zeta potential of the surface, which is traditionally assumed to match the electrostatic potential at the position where the velocity profile vanishes. This interface transport, which originates within the Debye layer at the interface, quantifies the width of the interfacial regions at the charged surface. The Debye layer, of size, λ_D_, results from the competition between ion attraction at the charged surface and the entropic effects. The value of λ_D_, is generally function of the salt concentration in the fluids (λ_D_ is about 3–4 nm for a concentration of salt of 0.01 M). The interfacial Debye layer is electrically charged, while the remaining bulk solvent is electrically neutral. Therefore, when applying the stress, the electric driving force is located within the Debye layer only. The driving force per unit surface acting on the liquid thus becomes Fe = σE, with σ the charge of the Debye layer, which is exactly opposite to the surface charge σ = εV_0_/λ_D_ for weak surface potential V_0_. This leads to Fe ~ −ε(V_o_/λ_D_)E. Now, the fluid velocity results from the balance between this electrical driving force in the Debye layer and the viscous stress at the surface. For a no-slip boundary condition, the latter is F_η_ ~ ην_E0_/λ_D_, since the velocity varies on a scale given by the Debye length. If slippage is exhibited at the surface, the velocity varies on the size λD + b and the viscous stress becomes accordingly F_η_ ~ ην_E0_/(λ_D_ + b), lower than the no-slip boundary conditions. Gathering these results, we get:

2 $$v\frac{{v}_{EO}}{{\lambda }_{D}+b}\approx -\,\varepsilon \frac{{V}_{0}}{{\lambda }_{D}}{E}$$

This leads to the Smoluchowski formula and provides an expression for the zeta potential in the form:

3 $${\rm{\zeta }}={{\rm{V}}}_{0}\,(1+{\rm{b}}/{{\rm{\lambda }}}_{{\rm{D}}})$$

Equation 3 shows that for a non-slipping surface, V_0_ can be identified as the potential at the plane of shear V0 = V(_Zs_), with Zs being of the order of one liquid layer. As expected, a strong amplification of charge transport is therefore demonstrated on a slipping surface. Generalizing this behavior to all interfacial transport phenomena, the flow fluid velocity in confined media results proportional to the applied gradient (stress, electrical potential) and depends on the size L, of the interface where the fluids interacts specifically with the mineral surface. Since our observed phenomenon is proportional to the applied stress that produces a surface energy gradient, we propose that slippage amplifies the transport according to the ratio b/L, which in turn controls the interfacial transport of fluids at the micro and nanometer scale. While structural defects at the external surface of minerals have been identified as limiting steps in transport and reactivity in non-confined fluids^32^, fast movement of CO_2_-rich fluids are related to fluid-fluid and fluid-calcite interfaces.

## Discussion

Our unexpected findings have potentially far-reaching implications for developing new fluid transport prediction models. Traditional approaches to fluid transport assume the presence of inter-grain pathways in polycrystalline materials such as micro and nano-pore tubes^36,37^. Fluid transport is usually observed in poly-phase material but individual mineral grains can also exhibit clear indications of multi-path migration even when visible evidence of such paths is lacking. Quantifying this process at the micro-nanoscale for a mineral at standard external conditions, we show that fluid mobility is in reality much higher than previously assumed using current predictive models^8,38–40^. Our findings also suggest that mechanical stress plays a more significant role in fluid transport than previously assumed. Classical reasoning assumes local partition equilibrium at the fluid-solid interface, or, equivalently, a continuity of the chemical potential. Based on our observations and on the approach outlined above, it would seem more consistent to introduce a discontinuous flux across the interface. The magnitude of this discontinuity characterizes the kinetics of pressure solution rather than intergranular diffusion. The rheological properties of small-size fluids and their flow in carbonate rocks are of paramount importance in many geological situations. In the case of lubrication of faults, calcite is an important, and in some cases dominant mineral in seismically active regions worldwide, leading an extra anisotropy in P- or S-wave velocities. Fast fluid movement at the microscale and nanoscale have significant consequences in interpreting anisotropy in fluid-bearing rock systems during carbon crust cycling^41–43^ and geological CO_2_ sequestration^17,44^. High mobility of fluids in crystals may also have important implications for fluid-mediated metal transport. The origin of native gold grains observed at the surface of sulfide grains in orogenic-type deposits^45^ could be explained by movement of micron-size fluid inclusions commonly observed in sulfides rather than by dissolution/precipitation processes involving external fluids^46^. Relatively low-strain (lower than Shear Modulus) could easily trigger micro-fluids containing dissolved gold (and other metals) to migrate out of the crystals, precipitating native metal like gold on the surface with no deformation of the parent sulfide minerals^47^, thus explaining field-scale petrographic observations.

## Methods

### Fluid inclusion analysis

The fluid inclusions were identified by preparing wafers of 150 µm thickness from the calcite crystals, polished on both sides to optimise transparency. The fluid composition was estimated non-destructively, by measuring the temperatures of phase change inside the fluid inclusions, using a Linkam THMGS 600 heating-freezing stage and following the procedures in Roedder (1984) and Shepherd *et al*. (1985). The stage was calibrated using synthetic material to accuracies of ±0.1 °C in the temperature range covered in this study. Salinity (expressed as wt.% eq. NaCl), bulk composition, and density data were calculated by reducing raw thermometric data with the BULK and ISOC software packages of Bakker)^48^. The equations of state used was that of Bowers & Helgeson (1983) for the H_2_O–CO_2_–NaCl system.

### Experimental Details

Atomic Force Microscopy (AFM) experiments were carried out under controlled atmospheric conditions with 70% residual humidity, using a Digital Instruments Nanoscope III device. In order to eliminate the possibility of residual dust dissolution in the water film on the calcite surface (cf. below), freshly cleaved grains were cleaned with a N_2_ flux just before experiments. Therefore, in order to avoid any interaction between tip and surface, the normal force applied to the samples was set to lower than 10 nN. We carried out all standard tests for imaging calcite samples: rotation, scaling, reproducibility on other sites and samples.
